# The Utility of a Pediatric COVID-19 Online Forward Triage Tool in Switzerland

**DOI:** 10.3389/fpubh.2022.902072

**Published:** 2022-07-07

**Authors:** Janet Michel, Annette Mettler, Carl Starvaggi, Nicola Travaglini, Christoph Aebi, Kristina Keitel, Thomas C. Sauter

**Affiliations:** ^1^Department of Emergency Medicine, Inselspital, Bern University Hospital, University of Bern, Bern, Switzerland; ^2^Emergency Telemedicine, University of Bern, Bern, Switzerland; ^3^Pediatric Emergency Medicine, Department of Pediatrics, Inselspital, Bern University Hospital, University of Bern, Bern, Switzerland; ^4^Department of Pediatrics, Inselspital, Bern University Hospital, University of Bern, Bern, Switzerland

**Keywords:** children, COVID-19, Coronavirus, utility, digital triage, expectation management, lessons learned

## Abstract

**Background:**

To offset the burden on the health system, hospitals set up telehealth interventions, such as online forward triage tools (OFTT). The website www.coronabambini.ch was developed to specifically address the needs of children and their families in Switzerland and to facilitate the decision to test, isolate, attend school, or access the health care system.

**Methods:**

Video interviews were held with key informants (*n* = 20) from a population of parents, teachers, guardians, as well as doctors who had used the child-specific COVID-19 OFTT and had consented to a further study. Convenience and quota sampling were done to include a variety of key informants. Interviews were recorded, transcribed verbatim, and analyzed for themes.

**Results:**

Three main themes emerged: i) the usefulness of the OFTT to the users, ii) expectation management and importance of stakeholder involvement in OFTT development, and iii) OFTT limitations.

**Conclusion:**

Our study highlights opportunities, limitations, and lessons to consider when developing a pediatric COVID-19 OFTT. The involvement of stakeholders, parents, teachers, and health care providers in the design, set up, implementation, and evaluation of telehealth interventions is critical as this can help with expectation management and enhance OFTT utility.

## Background

The first COVID-19 case in Switzerland was recorded on the 25 February 2020. A cloud of apprehension and fear engulfed the nation and the world, not knowing what to do and how to respond (1). The Swiss Federal Office of Public Health (FOPH) published pediatric COVID-19 testing recommendations to help families, caregivers, and health care providers with decisions regarding COVID-19 testing, isolation, and school and day-care attendance (2–5). These guidelines were complex due to the different age groups involved and age-specific symptoms, and it presented challenges not only to parents and caregivers but also to healthcare professionals. To facilitate the communication and implementation of the FOPH pediatric COVID-19 testing guidelines, Inselspital developed and provided free of charge, an online forward triage tool (OFTT), www.coronabambini.ch, which was available from 15 October 2020, and from December 2020 onwards it got integrated into the general COVID-19 OFTT provided by the FOPH (6).

During the COVID-19 pandemic, a variety of OFTTs were implemented and evaluated in the adult context (7, 8). Faced with a potential health system overload, rapidly evolving knowledge about a new virus, uncertainties in the population, specifically about the role of children within the pandemic, rapidly changing or complex guidelines, and public health recommendations, OFTTs can act as information sources, triage patients, and assist with public health communication (7).

In contrast to adult OFTTs, the environment and social contexts play a more prominent role in pediatric OFTTs. A myriad of factors influence how individuals and families follow or disregard tool recommendations, consequently affecting the OFTT utility. These factors include, but are not limited to, the severity of the child's illness, the family environment, and child care arrangements.

The purpose of this study is to assess the utility and usability of the pediatric COVID-19 OFTT www.coronabambini.ch, as well as to elicit reasons for adherence or non-adherence with the overall goal of providing recommendations to improve future OFTT development.

## Methods

### Context and Intervention

The Pediatric Emergency Medicine, Department of Pediatrics, Inselspital, Bern University Hospital, University of Bern, Switzerland and the Department of Pediatrics, Inselspital, Bern University Hospital, University of Bern together with the Department of Emergency Telemedicine at University of Bern developed and maintained an online forward triage tool (OFTT). The aim of www.coronabambini.ch was to help with the dissemination and interpretation of the pediatric COVID-19 testing guidelines published from the federal office of public health (FOPH) with regards to testing, isolation, quarantine, and school or day-care attendance. Development, structure, and usage of the OFTT have been published elsewhere (6).

### Study Design and Aim

We conducted an exploratory qualitative study with the aim to assess the utility of the pediatric COVID-19 OFTT.

## Central Question

Why did you use the OFTT and how useful was it to you?

### Sub Questions

What information did you search for but did not find?

How did you feel after consulting the online digital tool, with regards to fear, anxiety, and decision making?

What do you suggest needs to be done to make such an online tool more accessible and useful?

How can online tools like www.coronabambini.ch be adapted to make your decision-making processes easier?

Would you use the tool in a future pandemic and why or why not?

#### Sampling and Sampling Size

A purposeful and quota sampling approach was employed. Key informants (*n* = 20) were selected from the users of www.coronabambini.ch that consented to the study. The key informants included parents, teachers, doctors, and guardians of all sorts (Table 1). In qualitative research, saturation guides sample size and we aimed for both rich narratives and thematic saturation (9, 10). Saturation was reached by the 12^th^ Key informant (See Table 1).

**Table 1:** Key informants.

**Guardian Roles**	**Females**	**Males**	**Total**
Mother role	10	-	10
Mother plus health care professional, teacher	2	-	2
Father and medical professional	-	2	2
Father role		4	4
Father, Medical professional and or school authority		2	2
Total	12	8	20

### Data Collection and Analysis

Video rather than face-face interviews were held because of the pandemic situation to minimize COVID-19 risks and protect the health of all parties. Interviews were held between 7 June and 24 June 2022. An interview guide was used and adapted iteratively. Two researchers were present in each session and fielded the questions in turns. Interviews were recorded, transcribed verbatim, and analyzed. We used an inductive and deductive approach to data analysis and coded the interviews with a framework derived from other COVID-19 OFTT utility evaluation (11) studies while remaining open to new themes. Data management and analysis was done with MAXQDA2020 (VERBI software, Berlin).

#### Measure to Ensure Trustworthiness of the Data

Data collection and analysis were performed Iteratively, continuously adapting the interview guide to ensure dependability. The two qualitative researchers debriefed at the end of each interview and kept reflexive journals. To ensure transferability, a thick description of participants, context, and data collection process have been outlined.

### Ethics Approval

The local ethics committee of the Canton of Bern, Switzerland deemed our study exempt from full ethical approval (REQ-2020-01179).

## Results

Three main themes around the utility of the pediatric COVID-19 OFTT emerged namely; i) the usefulness of the OFTT to the users ii) expectation management and importance of stakeholder involvement in OFTT development, and iii) OFTT limitations (See Table 2).

**Table 2:** Summary of emergent themes.

**Theme**	**Category**	**Unit meaning**
Utility of OFTT	Information source	-support of authorities
	Allaying fear and anxiety	-symptoms, do not test
	Potential role in forward transmission reduction	-stay at home
	Potential role in health systems burden	-online access to info and recommendations
	reduction Decision making aid	-to test, isolate, seek care, send kid to school or not
Expectation management	Legitimacy	-teachers, use coronabambini
		-paediatricians-use coronabambini
		-parents, I have used coronabambini
	Testing recommendation	-buy in, paediatricians refusing to test kids after tool recommendation
		-parents not testing kids on purpose
		kids saying yes to all symptoms
	Expectation management	-diagnosis, QR code
		-parental instinct role
		-stakeholder involvement, paediatricians, parents, guardians
Limitations of tool	Symptom based OFTT	-new variants,
		-suggestibility of kids saying yes to all symptoms
		-infants-inability to express symptoms
		- tool accuracy and trust
	OFTT is an OFTT	-human factor, general practitioner (GP) or paediatrician
	Complex decision making	-social determinants, child care arrangements
	Accountability issues	-user/OFTT providers, who is responsible when things go wrong
		-no medical protection

### Theme 1 Utility of the OFTT to the Users

**Figure 1:**
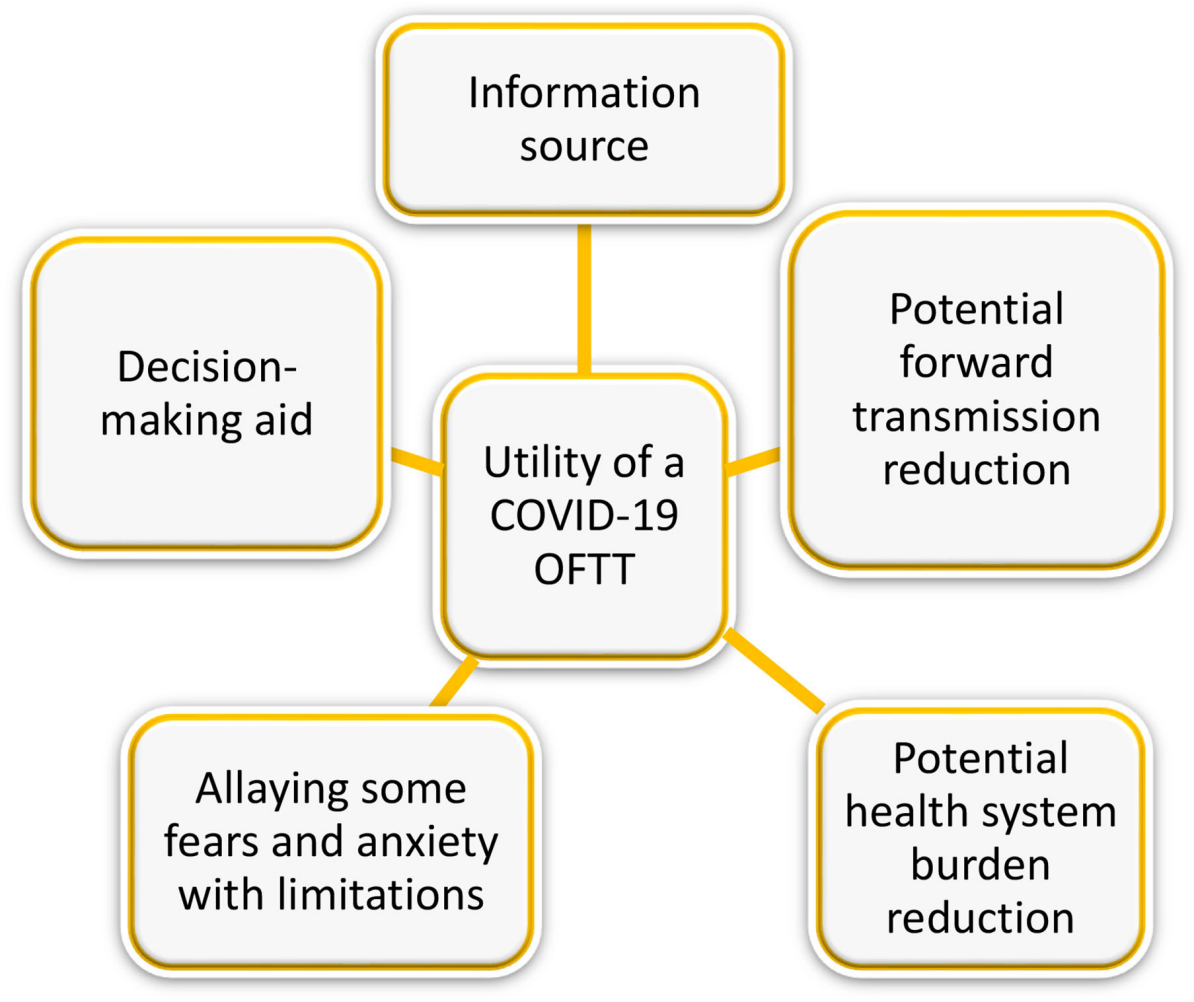
Utility of a COVID-19 OFTT.

Many key informants cited the usefulness of the OFTT as an information source in allaying some fears and anxiety to a certain extent, and as a decision-making aid. The information the parents wanted ranged from signs and symptoms to what to do when a child has come into a contact with a positive person, and the decisions they needed to make included, whether or not to visit the grandparents, keep the child at home or send the kid to school or day care. See also Figure 1. Below is what was said;

“*As parents we wanted to protect the kids from infection because we did not know the long-term effects on the heart, lungs and brain etc and that fear was present throughout. There was the immediate fear of infection and long-term fear of what could come in the future. The tool could not allay such fear*.” [Key Informant 2]

### Theme 2 Expectation Management and the Importance of Stakeholder Involvement in OFTT Development

#### Legitimacy Issues

The novel nature of SARS-CoV-2 created an information and knowledge gap. Parents, schools, and day cares grappled with what to do. They were all happy to find a child-specific tool recognized by FOPH that provided recommendations. The recommendations gave pediatricians, parents, and teachers legitimacy, sometimes against each other. The following was revealed:

“*Coronabambini gave me legitimacy as School committee head as some parents wanted that I do more and others wanted me to do less*.” [Key Informant 11]

“*That gave me legitimacy-when the teachers called and say are you sure the kid should be in school. I said yes, I have used coronabambini*.” [Key Informant 15]

#### Testing Recommendation and Testing Experience

One of the tool functions was to recommend parents to test or not to test their kid for COVID-19. The testing experience itself emerged as a major obstacle to following tool recommendations. Many key informants narrated a negative testing experience, marked by pain. One key informant told of her son's experience with the first test. The son experienced the test as painful and went on to share his testing experience with others at school. According to key informants, many kids who tested then refused subsequent testing. Many parents also revealed not to have tested their kids on purpose as revealed below;

“*Being sick is uncomfortable enough for a child hence a test should not exacerbate this discomfort. My 5-week-old baby‘s testing experience was a traumatic experience for me. I still see nurses holding the baby down and poking into her nose*.” [Key Informant 8]

#### Role of Parental Instincts

Many parents felt that the role of instincts needs to be acknowledged and incorporated in such OFTTs. In addition to the recommendations, key informants suggested including a clause in the tool stressing that parents use their discretion since an online tool remains an online tool. Are OFTT recommendations prescriptive or are they to aid parents in decision making? The fine line emerged as difficult to delineate. Some said the following;

“*As a mom, I do not always follow coronabambini recommendations, I use my own discretion, as a mother that knows her children well, the older one is good at simulating illness, the younger one not so*.” [Key Informant 15]

“*Coronabambini should take parents gut feelings into account e.g., when a kid is very sick even if it is negative, the child should stay at home. There are many questions raised for example where does individual responsibility end and when does communal responsibility to protect others kick in in a school setting?*” [Key Informant 11]

#### The Recommendation that Annoyed Many

Some parents expected to get a QR code after using the OFTT and others expected a diagnosis. For many, one recommendation did not go down well with them as revealed below;

“*I was disappointed by tool since it said call the doctor. This was a waste of my time answering all those questions. I could have done this myself from the outset. In the next pandemic I will call the doctor straight away*.” [Key Informant 16]

“*For us it was even more difficult that the pediatrician was not immediately available and so we had to call the emergency room*.” [Key Informant 10]

### Theme 3 Limitations of OFTTs

#### Symptoms Based OFTT

A symptom checker relies on the input of experienced symptoms to arrive at a recommendation. The process is rather cumbersome and tricky when children are involved. Key informants revealed that children are very suggestible, when asked, do you have a headache, the tendency is to affirm, leading to spurious recommendations. The process becomes even more complicated when an infant, who cannot express what he or she is feeling, is involved. The symptoms changed with new variants. The slight runny nose as the only symptom, got a recommendation not to test at the beginning of the pandemic before the emergence of newer variants. Many key informants disregarded this recommendation, only for the kid to test positive. Below is what one key informant said in this regard;

“*I don't even know. I'd say, if there were a homepage or a link to a homepage where you could or could have looked at the whole situation, Corona evolution with a child focus-that children could also spread the disease. I found it very difficult or there was sparse information about the children, what Corona is doing with them. Maybe that would be a helpful option, but I know it's always difficult, it always depends on the situation. I have no idea whether, my children's hospital did a few studies with schoolchildren. But whether the information is then clearer as to what to do or whether one is then even more confused, that will again depend on the user*.” [Key Informant 10]

##### Tool Accuracy and Trust

As reported above, the Alpha and Delta variants were marked with some changes in presenting symptoms. These differences stirred up issues of trust in the tool, associated with the recommendation not to test, which some parents disregarded. The COVID-19 tests done by some of these parents that disregarded the recommendation not to test came out positive, raising issues of OFTT reliability and validity. When probed if trust in the tool has been broken, many parents were understanding, citing the novel nature of the virus and the lack of prior knowledge as understandable. The availability of the quick tests also became a gamechanger, with some parents saying in future they would test first before using such a tool.

#### Accountability

Parental instincts and disregarding of tool recommendations reported above raise another issue; accountability when things go wrong. If a tool recommends the parent to test the child and they do not and send them to school, leading to a small outbreak, who is accountable? The importance of the pediatrician or primary care provider (PCP) was underlined. Many key informants felt that an OFTT is still an OFTT and these cannot replace the human component, PCP, or pediatrician, hence many also called a telehealth provider after OFTT use to get reassurance. Below is what one key informant said;

“*For us it was even more difficult that the pediatrician was not immediately available and so we had to call the emergency room*.” [Key Informant 10]

#### Complexity of Decision-Making Surpasses Capacity of OFTT

**Figure 2:**
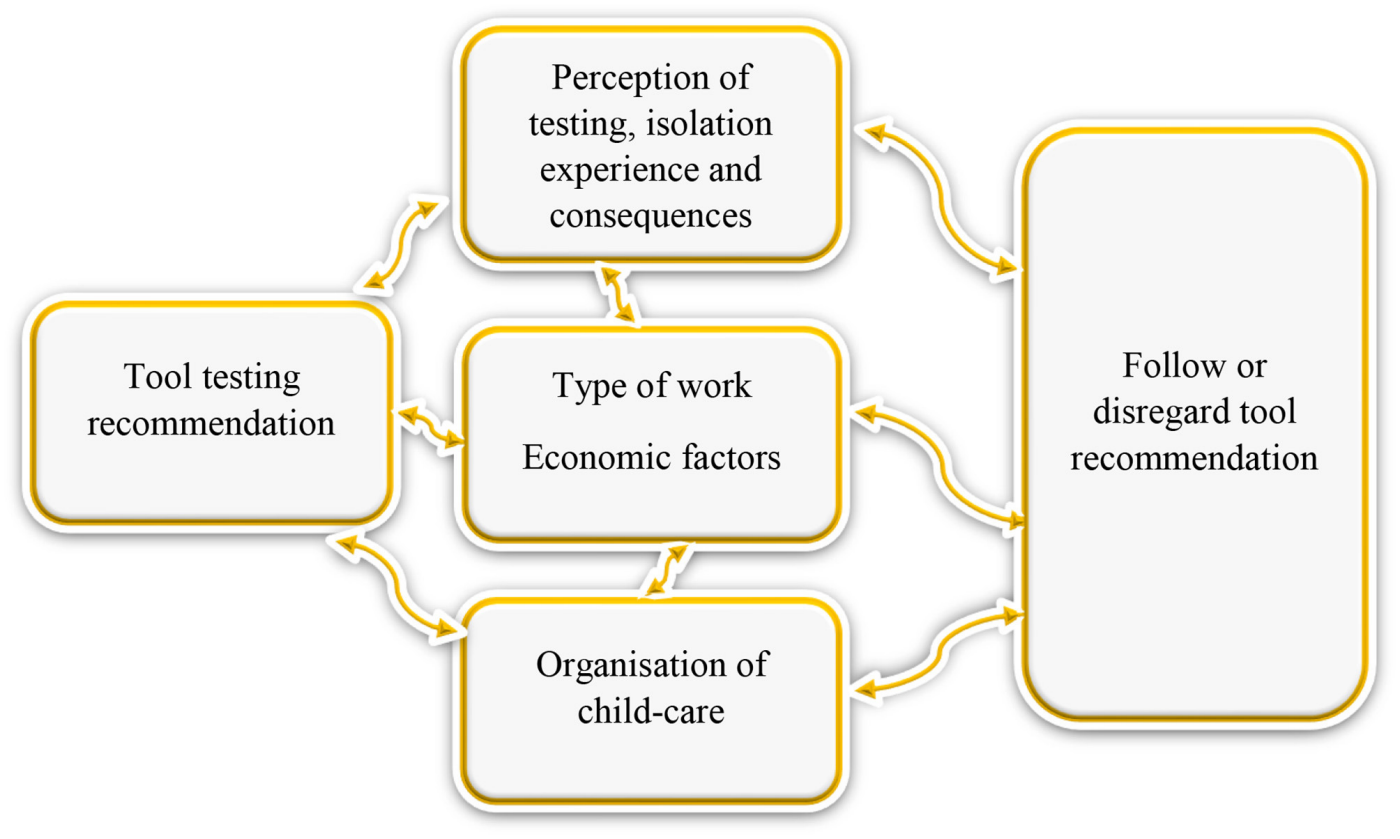
Complexity of decision-making regarding adherence to OFTT testing recommendation.

When asked why they used the tool, key informants revealed the following types of usage: to decide if to send the child to school/day care or not, to go to ER/Pediatrician, or not to visit the grandparents or not. Parents used the tool for both practical and medical decision-making. However, many other factors affected this decision that cannot be addressed by an OFTT, including, the type of work parents do, living space, home office possibility or not, revealing a myriad of factors that play a role in following or disregarding tool recommendations (See Figure 2, Table 2).

Noteworthy and closely related to the utility of the tool are the issues of accessibility and trust in the tool. The integration of the pediatric OFTT within the FOPH website made the tool accessible and also won the trust of the public.

## Discussion

With regard to the question of the utility and usefulness of the pediatric COVID-19 OFTT, three main themes emerged from our study; i) the usefulness of the OFTT to the users ii) expectation management and importance of stakeholder involvement in OFTT development, and iii) OFTT limitations (See Table 2, Figure 2).

### Utility of the OFTT to Users

In line with our findings, OFTTs have been found useful as an information source for symptoms and how to self-care and manage symptoms (12–14). According to literature, OFTT's main priority is to educate and inform the user (14). Our study also revealed the potential of the OFTT in allaying fear and anxiety to a certain extent. The fear of the unknown, particularly fear a novel infection, could not be addressed. Many key informants had questions like, “What will happen to my child in future? Will my child experience stunted development and more complications?” In line with our findings, there have been parallel pandemics, syndemics, one of which is fear (8). The role of telehealth in managing fear and anxiety warrants further research. There were indications that OFTTs have the potential to reduce forward transmission and health system burden. Our study could not quantify this but evidence in this regard has been reported elsewhere (15). By staying at home, many people avoided medical practices and hospitals, potentially reducing the forward transmission of the virus and health system burden (16).

Contradicting our findings, a study elsewhere revealed OFTTs as risk-averse, encouraging users to seek care where self-care was reasonable (17, 18). The key informants further found the tool useful as an aid in both practical and medical decision making (to send the child to school or not, to test or not, to seek emergency care or not). Further analysis revealed how complex this decision-making process is as it is influenced by a myriad of non-medicalfactors (19–21). The importance of socio-economic status and support networks, including the role of grandparents in child care, type of profession, living space, job, and income security emerged as influencing the decision-making process and consequently the decision to follow or disregard the tool recommendations. Our study revealed interconnectednesses that cannot be addressed by OFTTS and hence affect the usefulness of OFTTs in general and telehealth in particular. Social determinants of health, the conditions in which people are born, grow, work, live, economic policies, systems, and social norms among others, have an influence on health behavior (20, 21). These findings call for systems thinking in telehealth (22–26).

### Management of Expectations

Many key informants expected a diagnosis, which was not part of the OFTT objectives. What is an OFTT? What does it do? What output should one expect? Some recommendations emerged as not well received. The recommendation, “call your doctor the next day,” annoyed many key informants, who felt their time had been wasted by clicking through, inputting symptoms, only to be told to call their primary care provider (PCP). The FOPH testing algorithm purposefully recommended to call the PCP in the majority of situations as it was felt that PCPs should continue to function as gatekeepers in the pandemic. However, PCPs were sometimes difficult to reach and parents were frustrated. These findings point at the need to manage expectations by stating in the introduction what the OFTT purpose is, and what it can and cannot do. In line with our findings, patient expectations are likely to evolve as they get used to telehealth, and how telehealth providers respond to these expectations is critical (27). More work is needed to manage patient expectations (28). The involvement of end-users, the patients, and the health care providers (implementers) in the design, implementation, and evaluation of telehealth interventions can therefore not be over-emphasized.

### Limitations of OFTTs

The symptoms varied with the emergence of variants, affecting the validity and reliability of the recommendation to test. This emerged as a challenge with the potential to affect tool utility and trust in the tool. Some parents called for the role of parental instincts to be acknowledged and built in such telehealth tools-OFTTs. Closely linked to this was the finding that some health care providers, particularly pediatricians refused to test children despite the OFTT recommendation to test. This underscores the importance of involving both users and health care providers in the design, implementation, and evaluation of telehealth tools. The evolving virus, presentation, and guidelines made it even more challenging for the telehealth providers, highlighting, the importance of ensuring sufficient human, IT, and material resources when setting up OFTTs.

Of concern to many, was the fact that OFTTs are online tools. OFTTs cannot talk to, touch, feel, or look a patient in the eye. A vital diagnosing element, human conversation is missing, (14, 15) concurring with our findings. Closely associated with the above concern is the issue of accountability. Though our OFTT literally implemented the FOPH guidelines, the accountability between the OFTT and the testing recommendation itself was not fully clear to users. Many key informants raised the issue of the doctor–patient relationship. The human factor is still valued by many.

## Lessons Learned

In terms of whether a COVID-19 OFTT is useful and how this usefulness could be further enhanced for future OFTTs, our study revealed the following learning points:

Health care providers' attitudes affected tool utility, hence we recommend the involvement of health care providers and users in the design, implementation, and evaluation of telehealth tools.

Tool accessibility, usefulness, and trust depend on the source of tool and health authority support and recognition. It is advisable to work with and seek the support of recognized authorities to improve tool utility.

The general fear of the unknown remained even after OFTT use. The role of telehealth in managing fear and anxiety warrants further research.

Non-medical factors including socio-economic and testing experiences all affect adherence and consequently OFTT utility. Systems thinking in telehealth can therefore not be over-emphasized.

Patient expectation management is closely linked to trust. How telehealth providers respond to these expectations is critical (27), particularlyacknowledging the limits of and integrating OFTTs in the broader health care system and not offering them as standalone solutions. Key informants indicated that an online tool remains an online tool.

The evolving virus also meant evolving presenting symptoms that in turn affected tool validity, reliability, and potentially trust. This highlights the need for continuous and regular updating of such tools, hence the resources needed to establish such an intervention and approach needs to be well thought out, planned, and funded.

### Strengths and Limitations

We reported findings from the evaluation of one of the first child-specific COVID-19 OFTT set up in Switzerland. Insights and lessons gained can be useful for future pandemics. Our study demonstrated the OFTT utility beyond being an information source. Our study can provide guidance for the development of OFTTs in this and future pandemics. Important points such as embedding the OFTT in the broader health system can help address pitfalls and assist in expectation management. In consultation with the authorities, the OFTT was taken offline during the course of the pandemic, as the number of cases and testing frequency in Switzerland had fallen sharply and the OFTT was therefore no longer useful. It is possible to use the OFTT again at any time during the next wave. It would also be a conceivable option to use an OFTT for the early detection of a next wave.

The evaluation of our OFTT and the insights gained have the limitation that they come from a specific health care system setting in Switzerland. This health care setting with its different responsibilities at the cantonal and national level is specific to Switzerland but also exists in some international settings such as Germany. Another limitation is that our study was done with users of a specific OFTT in Switzerland. Transferability to other OFTTs in other contexts might be limited.

## Conclusion

Our study highlights opportunities, limitations, and lessons to consider when developing and using OFTTs. Our study demonstrated the utility of an OFTT beyond being an information source. OFTTs can be useful in allaying fear and anxiety but have limitations. The involvement of stakeholders, parents, teachers, and health care providers in the design, set up, implementation, and evaluation of telehealth interventions is critical, as this can help with expectation management and facilitate OFTT utility. The interconnectedness of factors revealed in our study findings highlights the complexity, underscoring the importance of systems thinking in telehealth.

## Data Availability Statement

The datasets presented in this article are not readily available because Interviews cannot be shared openly since the participants were assured of anonymity and therefore cannot be shared publicly. Any requests can be sent to the corresponding author. Requests to access the datasets should be directed to janetmichel71@gmail.com.

## Ethics Statement

Ethics review and approval/written informed consent was not required as per local legislation and institutional requirements of the local ethics committee of the Canton of Bern, Switzerland.

## Author Contributions

JM, AM, CS, NT, KK, and TS were involved in the study design and data collection. JM and TS carried out the qualitative data analysis and wrote the first draft. JM, AM, CS, NT, CA, KK, and TS contributed to further drafts. All authors contributed and approved the final draft.

## Funding

This study presented is partly co-financed by the Federal Office of Public Health, Switzerland and the Swiss national science foundation (196615).

## Conflict of Interest

The authors declare that the research was conducted in the absence of any commercial or financial relationships that could be construed as a potential conflict of interest.

## Publisher's Note

All claims expressed in this article are solely those of the authors and do not necessarily represent those of their affiliated organizations, or those of the publisher, the editors and the reviewers. Any product that may be evaluated in this article, or claim that may be made by its manufacturer, is not guaranteed or endorsed by the publisher.
