# Supplementary material for: The Utility of a Pediatric COVID-19 Online Forward Triage Tool in Switzerland
Source: Front Public Health. 2022 Jul 7;10:902072. doi: 10.3389/fpubh.2022.902072 (PMC9301458; doi:10.3389/fpubh.2022.902072)
Supplement: Supplementary file 1 [file Data_Sheet_1.pdf]

Annexure 1 Interview Guide

Qualitative Interviews - English

Timeframe: **45-55 Minutes**

Project Introduction

Presentation of the interview team, goal of the interview and thanks for participation. Consent to video recording and use of anonymized data.

Rapport

O Who (of your children) developed symptoms / contracted COVID-19? -age, canton, test result_

O Please describe yourself and your family so that we can get an idea of who we are dealing with-who do you live with, pets etc.

Digital Competence

O Do you use online tools in general? (for what, how often)

O What do you understand by an OFTT? When do you as a parent use this?

O Have you made more use of online tools since the beginning of the pandemic?

Accessibility

O How did you come across coronabambini.ch? online search?)

O Was the Coronabambini online digital tool easily accessible for you? -explain why or why not ... (easy to find on the homepage, length of the tool, clear and easy to follow instructions, language, order)

Usefulness as a reliable source of information and decision making

O Which information or components would have helped you better?

O What do you suggest what needs to be done to make such an online tool more accessible and useful?

O How can online tools like Coronabambini be adapted to make your decision-making processes easier? (keep the child at home, send to school, isolate, test)

O If you have consulted Coronabambini - have you used it as an aid in medical decision-making or for everyday life (request - have you used Coronabambini for general medical decisions or for practical questions, such as sending the child to day care, the grandparents too visit or not)

O Did you follow the recommended advice? Why did you follow the advice? So that the child can go to school so that the child can stay at home, got confirmation from friends, paediatrician / doctor, influence of the media on your decision-making process, trust in the OFTT provider?

O What made you disregard the advice and recommendations? - severity of symptoms, change in condition, practical childcare issues, public health concerns, why not tested after recommendation, unavailability of tests, stigma)

O Were the children cared for by such a person at risk? What is your understanding of an at-risk person for you? Probe- what are the challenges, how do you deal with the topic

Medical Knowledge

O What is your understanding of the infectivity of children? - do they transmit the virus as often as adults, why?

Usefulness in determining socioeconomic status

O Which families had / have challenges in relation to testing children - children had special testing sites? (low-socio-economic status, migrants, why, time, cost)

O When you completed the questionnaire, you stated your socio-economic status. How do you describe your socio-economic position?

O What do you understand by the average socio-economic status in Switzerland and how do you assess that (what factors should we take into account when trying to asses this)

O How does socio-economic status influence the decision-making processes of coronabambini users-quarantine, test behaviour, etc., please explain with examples

Experience with illness and tests

O Did your child experience COVID-19 symptoms? -please explain

O Please describe 1) your child's COVID-19 testing experience 2) your own COVID-19 testing experience (access to test, discomfort of the smear, pain)

O Why in your opinion did people who were given the recommendation to test not do so? (is the test invasive, depending on age, school child test experience, fear of isolation, 10 days at home with young children, time, support, socio-economic problems and consequences, migrant families)

O How often are your children currently being tested for COVID (BAG recommendations) - What do you think about that? Is it too much, should they test less often than adults-your opinion?

Self-isolation

O Please explain in more detail how your quarantine experience for yourself, child and others- what worked and what didn't, e.g. For example, the need to go to work to protect others rather than your own needs to put others first, dilemmas, challenges, experiences with schoolchildren's isolation

O Have you, your child or people you know experienced stigma-explain

Prevention of onward forward transmission

O If you have consulted a family doctor or paediatrician; did you call before?

O How did your GP or paediatrician react when you told them you suspected your child may have COVID-19 and that you used an online tool?

Allaying Fear and anxiety

O Did you feel reassured after visiting coronabambini.ch? - describe how you felt after consulting the online digital tool, regarding fear and assurance confidence that everything would be fine.

O Were you afraid for your child, for your parents, for other children?

O If your fears have not been allayed, how did you deal with your fears? ask what has increased your confidence, what has dispelled your fears)

Recommendations

O What would you do in a future pandemic? -e.g., search for Inselspital online tool, use another tool

O Fear and anxiety have been identified as a parallel pandemic in society. Are there other strategies to alleviate anxiety and fear) that you find effective alone or in conjunction with online digital tools when faced with an epidemic like COVID-19 that is accessible to parents with children?

Outlook

O The road to recovery has been described by many as arduous and long - How was your child's, your own experience? any protracted problems, psychiatric or other aftereffects - explain.

O We have heard from other people that the pandemic has changed our society. What personal life lessons did you learn during this pandemic and changes do you foresee in the future? Society, health and health system, economy and future prospects

Annexure 2 Interview Guide Deutsch

Zeitrahmen: **45-55 Minuten**

Einführung

- Vorstellung des Interview-Teams, Ziel des Interviews und Bedanken für Teilnahme. Einverständnis Videoaufnahme und verwenden anonymisierter Daten.

Rapport

- Wer (von ihren Kindern) entwickelte Symptome/erkrankte an COVID-19?
  - Alter, Kanton, Testergebnis
- Bitte beschreiben Sie sich und Ihre Familie, damit wir uns ein Bild davon machen können, mit wem wir es zu tun haben.
  - Mit wem leben Sie zusammen, Haustiere etc.

Digitale Kompetenz

- Nutzen Sie Online-Tools im Allgemeinen? (wofür, wie oft)
  - Was verstehen Sie unter einer OFTT? Wann setzen Sie als Elternteil diese ein?
- Nutzen Sie Online-Tools vermehrt seit Beginn der Pandemie?

Verfügbarkeit

- Wie sind Sie auf coronabambini.ch gekommen? (hingewiesen durch ..., Online-Suche)
- War das digitale Online-Tool *Coronabambini* für Sie leicht zugänglich? Erklären Sie, warum oder warum nicht... (leicht auf der Homepage zu finden, Länge des Tools, klare und leicht zu befolgende Anweisungen, Sprache, Reihenfolge...)

Nützlichkeit als zuverlässige Informationsquelle und Entscheidungsfindung

- Welche Informationen oder Komponenten hätten Ihnen besser geholfen?
  - Was schlagen Sie vor, was getan werden muss, um ein solches Online-Tool zugänglicher und nützlicher zu machen?
- Wie können Online-Tools wie *Coronabambini* angepasst werden, um Ihre Entscheidungsprozesse zu erleichtern? (das Kind zu Hause behalten, in die Schule schicken, isolieren, testen)
- Wenn Sie *Coronabambini* konsultiert haben - haben Sie es als Hilfe bei der medizinischen Entscheidungsfindung oder für den Alltag genutzt (Aufforderung - haben Sie *Coronabambini* für allgemeine medizinische Entscheidungen oder für praktische Fragen genutzt, wie z.B. das Kind in die Tagesstätte zu schicken, die Großeltern zu besuchen oder nicht)
- Haben Sie die empfohlenen Ratschläge befolgt? Wieso haben sie die Ratschläge befolgt?
  - Damit das Kind zur Schule gehen kann, damit das Kind zu Hause bleiben kann; Fragen, Auswirkungen Bestätigung von Freunden, Kinderarzt/Arzt, Einfluss der Medien auf Ihren Entscheidungsprozess, Vertrauen in den OFTT-Anbieter?
- *Was hat Sie dazu veranlasst, die Ratschläge und Empfehlungen* ***nicht*** *zu befolgen? (Schwere der Symptome, Veränderung des Zustands, praktische Fragen der*

Kinderbetreuung, Bedenken der öffentlichen Gesundheit, warum wurde nach der Empfehlung nicht getestet, Nichtverfügbarkeit von Tests, Stigma)

- Wurden die Kinder von einer Risikoperson solche Person betreut? Was ist für Sie eine Risikoperson?
  - Aufforderung zum Verständnis der Risikoperson, was sind die Herausforderungen, wie gehen Sie mit dem Thema um

Medizinische Kenntnisse

- Was ist Ihr Verständnis über die Ansteckungsfähigkeit von Kindern? Übertragen sie das Virus genauso häufig wie Erwachsene, warum?

Nützlichkeit bei der Ermittlung des sozioökonomischen Status

- Als Sie den Fragebogen ausfüllten, gaben Sie Ihren sozioökonomischen Status an. Was verstehen Sie unter dem durchschnittlichen sozioökonomischen Status in der Schweiz und wie schätzen Sie diesen ein (Probe-Faktoren)
  - Welche Faktoren gehören für Sie zum sozioökonomischen Status?
  - Wie beschreiben Sie Ihre sozioökonomische Stellung?
  - Wie könnte der sozioökonomische Status den Entscheidungsprozess (Quarantäne-, Testverhalten etc.) beeinflusst haben.
- *Welche Familien haben Herausforderungen in Bezug auf das Testen von Kindern - Kinder hatten spezielle Teststellen? (niedriger sozioökonomischer Status, Migranten, warum, Zeit, Kosten)*

Erfahrungen mit Krankheit und Tests

- Hat Ihr Kind COVID-19-Symptome gehabt? Erklären Sie.
- Beschreiben Sie bitte 1) die Erfahrung Ihres Kindes 2) die Erfahrung, die Sie mit dem COVID-19-Test gemacht haben (Aufforderung zum Zugang zum Test, Unannehmlichkeit des Abstrichs, Schmerzen unangenehm
- Warum haben sich Leute, die die Empfehlung/Gelegenheit zum Testen bekamen, nicht getestet?
  - Ist der Test invasiv, je nach Alter, Schulkind-Testerfahrung, die Angst vor Isolation, 10 Tage mit kleinen Kindern zu Hause, Zeit, Unterstützung, sozioökonomische Probleme und Konsequenzen, Migrantenfamilien
- Wie oft werden Ihre Kinder derzeit auf COVID getestet (BAG-Empfehlungen).
  - Was sagen Sie dazu? Ist es zu viel, sollten sie weniger oft getestet werden als Erwachsene (Aufforderungen erläutern, warum)
- Selbstisolation. Bitte erklären Sie genauer, wie Sie das erlebt haben, was funktioniert hat und was nicht, z. B. das Bedürfnis, zur Arbeit zu gehen, um andere zu schützen, und nicht die eigenen Bedürfnisse, andere an die erste Stelle zu setzen, Dilemmas, Herausforderungen, Erfahrungen mit der Isolation von Schulkindern
- Haben Sie Erfahrungen mit Stigmatisierung - Menschen, die Sie und Ihre Familie meiden? (Konsequenzen des Testens)

Nutzen zur Vermeidung von Kreuzinfektionen

- Wenn Sie einen Haus- oder Kinderarzt konsultiert haben; haben Sie vorher angerufen?
- Wie hat Ihr Haus- oder Kinderarzt reagiert, als Sie ihm mitteilten, dass Sie den Verdacht haben, dass Ihr Kind COVID-19 hat und dass Sie ein Online-Tool benutzt haben?

Nutzen zur Linderung von Angst und Befürchtungen

- Beschreiben Sie, wie Sie sich nach der Konsultation des digitalen Online-Tools gefühlt haben, in Bezug auf das Gefühl von Angst und oder Zuversicht, dass alles gut werden würde? (haben Sie sich nach dem Besuch von coronabambini.ch beruhigt gefühlt)
  - Hatten Sie Angst um Ihr Kind, um Ihre Eltern, um andere Kinder?
- Wenn Ihre Ängste nicht zerstreut wurden, wie sind Sie mit Ihren Ängsten umgegangen? fragen Sie nach, was Ihre Zuversicht gestärkt hat, was Ihre Ängste zerstreut hat)

Empfehlungen

- Was würden Sie bei einer zukünftigen Pandemie tun? (z.B. Online-Tool des Inselspitals suchen, ein anderes Tool verwenden)
- Furcht und Angst wurden als eine parallele Pandemie in der Gesellschaft identifiziert. Gibt es andere Strategien (um Angst und Furcht zu lindern), die Sie allein oder in Verbindung mit digitalen Online-Tools für wirksam halten, wenn Sie mit einer Epidemie wie COVID-19 konfrontiert sind, die für Eltern mit Kindern zugänglich ist?

Ausblick

- Der Weg zur Genesung wird von vielen als sehr mühsam und lang beschrieben - Wie war Ihre Erfahrung? Haben Sie irgendwelche langwierigen Probleme, psychiatrische oder andere Nachwirkungen erlebt - erklären Sie das.
- Wir haben von anderen Personen gehört, dass die Pandemie unsere Gesellschaft verändert hat. Welche persönlichen Lebenslektionen haben Sie während dieser Pandemie gelernt und welche persönlichen Veränderungen sehen Sie für die Zukunft voraus?
  - Probe. Gesellschaft, Gesundheit und Gesundheitssystem, gesellschaftliche (sozioökonomische) und zukünftige Aussichten
